# Seroprevalence of anti-HEV IgG in children: very early exposure in young children in a hyperendemic region

**DOI:** 10.3389/fpubh.2023.1293575

**Published:** 2023-11-13

**Authors:** Lisandru Capai, Shirley Masse, Nathanaël Hozé, Dorine Decarreaux, Jean Canarelli, Marie-Hélène Simeoni, Xavier de Lamballerie, Alessandra Falchi, Rémi Charrel

**Affiliations:** ^1^UR 7310, Université de Corse, Corte, France; ^2^AG Junglen, Institute of Virology, University of Charité, Berlin, Germany; ^3^Mathematical Modelling of Infectious Diseases Unit, Institut Pasteur, Paris, France; ^4^Unité des Virus Émergents, Aix-Marseille University, Marseille, France; ^5^Laboratoire de Biologie Médicale, CCF, Ajaccio, France; ^6^Laboratoire de Biologie Médicale 2A2B, Corte, France; ^7^Comité de Lutte contre les infections Nosocomiales, APHM HOPITAUX Universitaires de Marseille, Marseille, France

**Keywords:** hepatitis E virus, serosurvey, hyperendemic area, children, epidemiology

## Abstract

**Background and objectives:**

Hepatitis E virus (HEV) can be considered an emerging zoonotic pathogen and is an important cause of acute viral hepatitis in high-income countries. Corsica has been identified as a hyperendemic region for HEV. We aimed to characterize the prevalence of IgG among children and estimate the annual force of infection of HEV.

**Methods:**

From April 2020 to June 2021, we collected 856 “residual sera” in 13 medical biology laboratories. Sera were tested using the Wantaï HEV IgG assay. Data were weighted according to the distribution by sex and age of the real Corsican population. Serocatalytic models were applied to assess the annual force of infection of HEV.

**Results:**

The weighted seroprevalence was 30.33% [27.15–34.0]. The seroprevalence was only associated with increasing age (7.25–40.52%; *p* < 0.001). The annual probability of infection was 5.4% for adults and children above 10-year-old and 2.2% for children under 10 yo.

**Conclusion:**

Our study demonstrates that in the hyperendemic island of Corsica, (i) exposure of the population to HEV is homogeneous at the spatial level with no difference between genders; (ii) HEV exposure occurs from birth, resulting in 7.4% seropositivity at the age of 4 years; and (iii) super exposure is observed after the age of 9 years. Accordingly, specific studies should be conducted to determine the breadth of the situation identified in our study. The role of the environment and its contamination by domestic or wild swine excreta should be investigated using a One Health approach.

## Introduction

In recent years, a sharp increase in hepatitis E virus (HEV) infections has been reported in Europe (1), and the disease has become a serious public health problem in high-income countries (HICs). Most cases are caused by genotype 3 (HEV-3) and genotype 4 (HEV-4) through zoonotic transmission mainly associated with consumption of raw or undercooked pork meat (2, 3).

In HICs, the prevalence of HEV infection is globally lower than in low- and middle-income countries (LMICs) (4). However, in some geographic areas such as Central Italy, the Netherlands, Southwestern France, and Poland, unexpectedly high seroprevalence rates allowed their qualification as hot spots or hyperendemic regions (5–11). Most studies were conducted in adults, and data on children are scarce (12). Interestingly, in hot spots such as Corsica (France), more than 40% of young adults (18–27 yo) display already high seroprevalence rates, showing that HEV infection could occur during childhood (13, 14). Among adults, we previously identified that skinning/evisceration of wild boars, consumption of pork liver sausages (Ficatellu, Fittone), hiking, and drinking fountain water in villages or natural spring water are significantly associated with higher seroprevalences (14). However, the kinetics of HEV infection during childhood has never been studied in HICs, although it may yield insight into the pathways of transmission that are likely to be at least partially different from the risk factors in adulthood.

Accordingly, we conducted a seroprevalence study in Corsica using residual sera from medical biology laboratories across the island. The main objective was to understand better the kinetics of HEV infection among children below 18 yo and to describe the shape of the curve to evaluate the different modes of transmission in hyperendemic regions.

## Methods

### Sampling plan

The necessary number of samples was calculated with the method described by Arya, Antonisamy (15). The calculated minimal number of samples from children was 653 in order to estimate a seroprevalence with a confidence level of 95% and a precision of 3%. This calculation took into account the actual population of children in Corsica (70,299 children between 0 and 19 yo in 2021) and a predicted seroprevalence of 30% (using the results of our previous study, the annual force of infection among children is 3.8%) (13).

### Samples, information collected, and regulations

**Figure 1:**
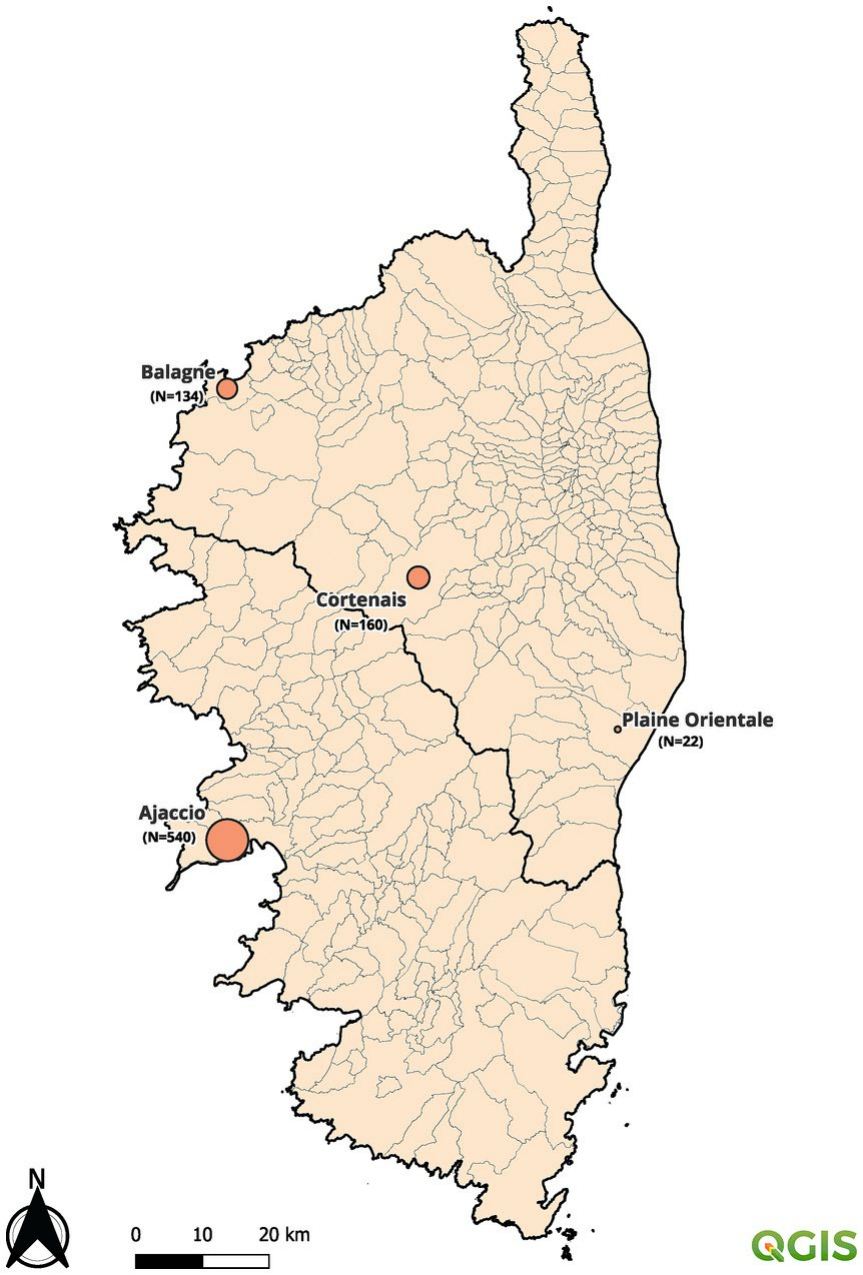
Map showing the location of laboratories, collection areas and number of samples.

From April 2020 to June 2021, we collected 856 “residual sera” in partnership with 13 medical biology laboratories located in Corsica [as previously described in (16); Figure 1]. After collection of the whole blood, the serum was obtained by centrifuging at 1,000–2,000 ×*g* for 10 min in a refrigerated centrifuge. All duplicates were excluded from the database, based on the criteria of date of birth, gender and laboratory. No other exclusion criteria was applied.

For each serum, age, sex, place, and date of collection were obtained anonymously in line with reference methodology MR-004 according to French law 2016–41 of January 26, 2016, on the modernization of the French health system. The authorizations were obtained on April 20, 2020 and the identification number of the study to the French Data Protection Authority (Commission Nationale de l’Informatique et des Libertés) is CNIL Reference: 2217594 v 0.

### Detection of HEV IgG

HEV IgG was detected using the Wantaï kit (Wantaï Biologic Pharmacy Enterprise, Beijing, PRC), which was also used as a reference test by the French reference center for hepatitis E. It is based on a recombinant antigen encoded by an ORF-2 region (17) and has been analytically and clinically evaluated (18). Blank, positive and negative controls were used as recommend by the manufacturers. All the serological tests were performed using the Multiskan GO spectrophotometer (Thermo Fisher Scientific). For each sample, a ratio (absorbance of the sample/cutoff) was calculated; values ≥1 were considered positive. The cut-off value corresponds to the Average of the Negative Control values ANC + 0.03.

### Statistical analyses

Descriptive statistical analysis was performed for age, gender, and geographic area. Age was described as median, mean, and interquartile ranges (IQRs). Categorical data were reported as percentages. HEV IgG seroprevalence and the 95% exact binomial confidence intervals (CIs) were estimated. The base was weighted according to distribution by sex and age of the real Corsican population (proportions observed in the general population were obtained from the French National Institute of Statistics and Economic Studies). Associations of the presence of HEV IgG with sex, age, and location were tested using the χ^2^ test or Fisher’s exact test. The odds ratio (OR) was used to describe the risk of sera being positive. Statistical significance was set at a value of *p* < 0.05. All statistical analyses were performed using R software version 3.6.1 (R Foundation, Vienna, Austria).

### Models of annual force of infection

The analysis of seroprevalence by age provides insight into the history of infection of HEV and the long-term immune mechanisms that lead to the antibodies’ decay. Serocatalytic models aim to reconstruct the annual force of infection (the rate at which susceptible individuals are infected) from the age profile of seroprevalence.

Here, a serocatalytic model was retained, in which the force of infection λ is age-invariant and immunity decayed with a rate ρ . The annual probability of infection is $p=1-e^{-\lambda }$ (19) and the probability for an individual of age a to be seropositive is given by (20):


$$P_{a}=\frac{\lambda }{\lambda +\rho }\left(1-e^{-\left(\lambda +\rho \right)\times a}\right)\text{.}$$

Here, we combined samples of the 856 children throughout Corsica with N = 1,094 samples from Corsica natives above 18 yo. We considered a model where the force of infection is constant in time and across the different sampling locations but differs for children under 10 yo and above, and where immunity decayed with a rate ρ . From the above formula, we derive the probability for an individual to be seropositive varies with age and is given for a≤10 by:


$$P_{a}=\frac{\lambda_{1}}{\lambda_{1}+\rho }\left(1-e^{-\left(\lambda_{1}+\rho \right)\times a}\right)\text{,}$$

and for age a>10 by:


$$\begin{array}{l} P_{a}=\frac{\lambda_{2}}{\lambda_{2}+\rho }\left[1-e^{-\left(\lambda_{2}+\rho \right)\times \left(a-10\right)}\right]+\\ \;\frac{\lambda_{1}}{\lambda_{1}+\rho }\;\left[1-e^{-\left(\lambda_{1}+\rho \right)\times 10}\right]\;e^{-\left(\lambda_{2}+\rho \right)\times \left(a-10\right)}\text{.} \end{array}$$

Here, $\lambda_{1}$ and $\lambda_{2}$ are the force of infection of individuals below and above 10 yo, respectively. The contribution to the likelihood of an individual of age *a* with seropositivity status *s* (*s* = 1 if seropositive; otherwise, *s* = 0) is ${\left(P_{a}\right)}^{s}{\left(1-P_{a}\right)}^{1-s}$ . Parameters were estimated in a Bayesian framework using a Markov Chain Monte Carlo method implemented in R using the Rstan package (21). Four independent chains of 10,000 iterations each were simulated, with the first 5,000 iterations corresponding to the burn-in. Flat priors were chosen for the force of infection $\lambda_{1}$ and $\lambda_{2}$ and the seroreversion rate ρ .

The posterior distributions’ mean and 95% credible intervals (CrI) were calculated. We report a susceptible individual’s annual probability of infection using the formula $p=1-e^{-\lambda }\text{.}$ The seroreversion rate was converted into a duration of immunity using the expression $d=\frac{1}{\rho }\text{.}$

To integrate the modeling results of our previous study among adults (13), we compared the different populations by age group.

## Results

Eight hundred and fifty-six sera collected between April 2020 and June 2021 were tested (Table 1). Overall, the mean and the median age were 13.4 years and 15 years, respectively (Min-Max: 1 month–19 years; IQR 11–17), 512 (60.7%) were female and 344 male (39.3%). Among the 856 sera, 280 contained HEV IgG (32.71%; CI 95 [29.57–35.85]). The HEV IgG weighted rate (sex and age) was 30.33% CI 95 [27.15–34.0] in the overall population (Table 1).

**Table 1:** HEV IgG seroprevalence of children and univariate analysis of variables.

	Raw data seroprevalences				Weighted seroprevalence by age and sex					
Variables	Nb. of sera	Nb. of positives	HEV IgG (%)	[CI 95%]	Nb. of sera	Nb. of positives	HEV IgG (%)	[CI 95%]	Value of *p* the variable	
Age groups	0–4	69	5	7.25	[2.39–16.10]	54.99	4.07	7.40	[0.95–13.84]	**<0.001**
	5–9	110	15	13.64	[7.84–21.49]	218.01	30.33	13.91	[7.35–20.47]	
	10–14	218	74	33.94	[27.67–40.64]	327.00	112.82	34.50	[28.10–40.90]	
	15–19	459	186	40.52	[35.99–45.17]	368.92	146.63	39.75	[34.77–44.72]	
Gender	Female	512	179	34.96	[30.83–39.26]	462.99	139.31	30.09	[25.69–34.48]	0.893
	Male	344	101	29.36	[24.59–34.48]	505.93	154.54	30.55	[25.52–35.56]	
Geographic area	Ajaccio	540	178	32.96	[29.00–37.10]	628.29	187.07	29.77	[25.61–33.93]	0.879
	Balagne	134	44	32.84	[24.97–41.47]	130.87	42.11	32.18	[23.29–41.07]	
	Cortenais	160	50	31.25	[24.16–39.04]	183.76	54.97	29.91	[22.15–37.68]	
	Plaine Orientale	22	8	36.36	[17.19–59.34]	26.00	9.69	37.27	[14.86–59.68]	
Overall	856	280	32.71	[29.57–35.85]	968.92	293.84	30.33	[27.15–34.0]		

Bold values: significant values (value of *p* < 0.05); Nb., number; HEV, hepatitis E virus; IgG, immunoglobulin G; CI, confidence interval.

### Seroprevalence of HEV IgG

#### Seroprevalence according to sex

Among the 512 samples collected from women, 179 were positive (34.96% CI 95 [30.83–39.26]), and the weighted seroprevalence was 30.09% CI 95 [25.69–34.48]. Concerning the samples from men (*N* = 344), 101 were positive (29.36% CI 95 [24.59–34.48]) and the weighted seroprevalence was 30.55% CI 95 [25.52–35.56]. No significant difference was observed (value of *p* = 0.893).

#### Seroprevalence by geographical area

The sera were collected in four geographic districts: 540, 134, 160, and 22 sera originated, respectively, from Ajaccio, Balagne, Corte, and Plaine Orientale. No significant difference was observed between the regions (value of *p* = 0.879).

#### Seroprevalence by age group

**Figure 2:**
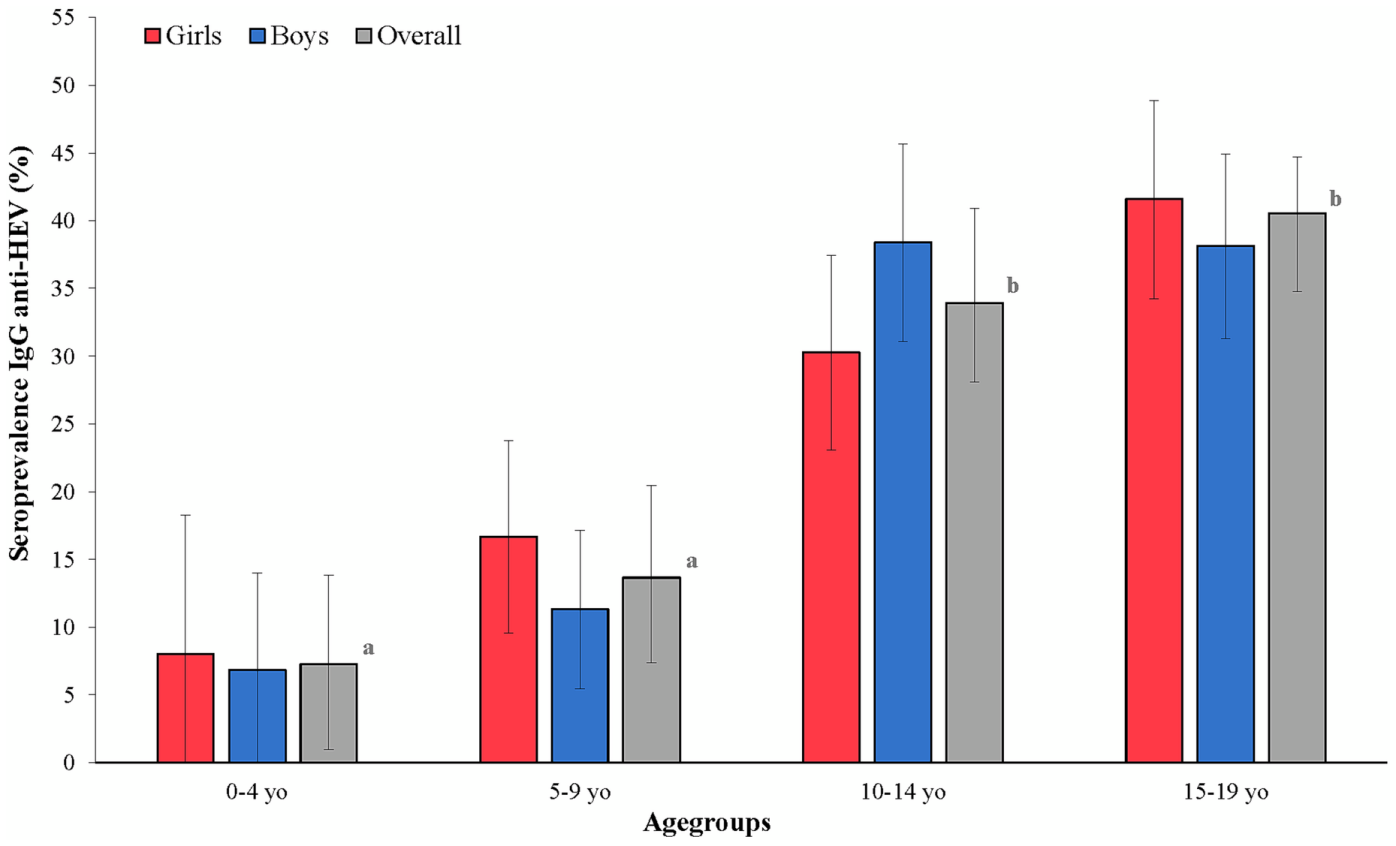
HEV IgG rates by age and sex among Corsican children. The different letters (a and b) mean a significant difference between seroprevalences by age groups in the overall population. The black bars represent the confidence intervals of 95% of the different seroprevalences.

Seroprevalence rates increased significantly with age and ranged from 7.40% CI 95 [0.95–13.84] in the 0–4-yo age group to 39.75% CI 95 [34.77–44.72] in the 15–19-yo age group (value of *p* < 0.001). Positivity rates were significantly different between the 5–9-yo age group (13.91% CI 95 [7.35–20.47]) and the 10–14-yo age group (34.50% CI 95 [28.10–40.90]) (Figure 2). The comparison of rates between the different age groups showed that the two youngest groups (0–4 yo and 5–9 yo) were not significantly different from each other (value of *p* = 0.25) but were significantly different from the two oldest classes (10–14 yo and 15–19 yo; value of *p* = 3.52 E-08) (Figure 2).

#### Models of the annual force of infection

To integrate the modeling results of our previous study, we compared the different populations (blood donors, university population, and patients sampled for biological analysis) by age group. None of the observed differences was significant, supporting the idea that exposure to HEV was similar. Therefore, we extended the zero catalytic modeling of adult blood donors in the seroprevalence study of Capai, Hoze (13).

**Figure 3:**
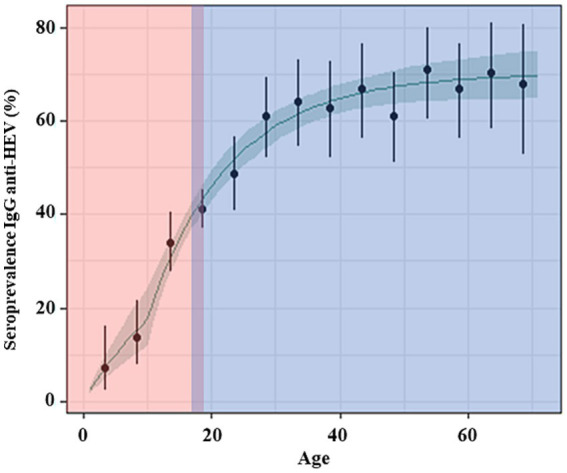
Observed and predicted profiles of HEV seroprevalence by age. The green line and green shadow represent, respectively, the mean and 95% credible interval of the seroprevalence predicted for a model of constant transmission with a higher risk of infection for children below 10 yo and with seroreversion. The black dots and bars are the point estimate and the 95% confidence interval of the seroprevalence in 5-year age groups. The red area is the data from this study in children, and the blue area is the data from our previous studies among adults.

The seroprevalence curve (Figure 3) showed a plateau (60–70%) in individuals in their late 20s, extending to the oldest age group. This stability might be related to either (i) a steady transmission (same infection rate) combined with a stable loss of immunity; or (ii) a model with a steady transmission and a higher risk infection for children. However, at the outset of this study, data for children needed to be improved. This precluded choosing one model over the other. Therefore, we combined children’s data with adults’ data, both native of Corsica, and implemented various serocatalytic models. As a result, the combined data are consistent with a model of constant transmission, where the annual probability of infection is 5.4% (95% credible interval, CrI: 3.7–6.9%) for adults and children above 10 yo and 2.2% (95% CrI: 1.5–3.0%) for children under 10 yo (Figure 3).

## Discussion

Few studies have addressed the rates of HEV antibodies in children living in HICs (United States, Turkey, Spain, Portugal, and Russia); all of those reported rates were < 6% (22–27). The unique study reporting high rates in children (34%) was performed in Southwestern France by the HEV Reference Laboratory using the same assay as used in our study. However, the cohort did not represent the regional population, as it consisted of hospitalized children (28). Furthermore, data for children living in other aforementioned hot spots (Central Italy, Poland, and the Netherlands) are necessary. Only one study has reported a high rate of HEV IgG in children, raising the question of the kinetics of HEV infection during childhood. A better understanding of the kinetics of HEV infection during childhood would help identify the role of different transmission pathways, whose impact and importance might be drastically different in children and adults, particularly those involved during infancy.

In our study, the global seroprevalence rate was shown to be eight to 10 times higher than rates reported in other studies in HICs (12, 22–27). Interestingly, the age-related gradient reported in non-hyperendemic areas was also observed in our study (12). Also, a significant sharp increase (>20%) is observed from the 5–9-yo class (13.6% [7.8–21.5]) to the 10–14-yo class of age (33.9% [27.7–40.6]). The strong increase around age 10 was also reported in Southwest France (14.4–28.6% between the 6–10-yo and 11–15-yo age groups) (28). Furthermore, we did not observe sex-based differences, which aligns with described results after age stratification and multivariate analysis in Corsica and Southwestern France among adults (28–32). Interestingly, subregional rates were similar, confirming that risk exposure is not regionally diverse in Corsica.

The analysis of seroprevalence stratified by age can provide key insights into the history of the circulation of HEV in Corsica as well as the patterns of long-term antibody decay following HEV infection. Indeed, serocatalytic models were used in our previous study (13) to reconstruct trends in the force of infection $\lambda_{a}$ , defined as the *per capita* infection rate of susceptible individuals of age a (33). As a result, we determined an important difference in the age-specific probability of infection: 5.4% for adults and children above 10 yo compared with 2.2% (95% CrI: 1.5–3.0%) for children under 10 yo.

In HICs, HEV is mainly acquired through pathways involving the swine reservoir, either by consumption of liver products or through direct contact with infected animals (8, 28, 32, 34–37). Moreover, many studies have shown that HEV persists in the environment (38–40). HEV was detected in drinking water (41), wastewater (42), surface water (42–44), water used for irrigating fruits and vegetables (45–48), and filter-feeding animals (49–51). A recent meta-analysis has estimated the prevalence of HEV in water in Europe at 12.2% (40). Exposure to HEV-contaminated water might therefore be riskier in HICs than initially believed. As in LMICs, exposure to contaminated water may represent a major risk factor in the epidemiology of hepatitis E. In support of this hypothesis, drinking bottled water was proven to be protective in Southwestern France (32). Concerning newborns, maternal antibodies can be transmitted either through breast milk or the placenta (52, 53). This transmission via the mother could also be at the origin of certain positive sera in the 0–4 age group, and therefore not linked to direct infection.

The shape of the curve reflecting the kinetics of infection shows that the studied populations have been exposed to HEV from birth. The increasing exposure observed after 10 years of life suggests another exposure risk that is superimposing on top of the initial one. Moreover, the plateau was explained by the fact that 50% of the infected individuals lose their immunity after 46 years (95% CrI: 27 years–87 years). Although there is a strong association between hyperendemic areas and the consumption of raw or undercooked pork meat (usually different types of dried sausages), children may be exposed to additional contamination pathways, such as those related to the environment. Exposure to undercooked pork meat is the superimposing factor that drives curve sharpening. By contrast, environmental exposure to different sorts of contaminated water is responsible for a steady exposure rate from birth to adulthood. Specific studies should be conducted for other hot spots, such as Central Italy, Poland, and the Netherlands, as the lack of data precludes any conclusion. Different interesting points were noticed when we compared the kinetics of infection in the other hot spots for HEV: (i) the seroprevalence rates in young adults (10, 19, 22.7, and 35% in the Netherlands, Southwestern France, Poland, and Central Italy, respectively) (5–11) were significantly lower than those determined in Corsica in our two previous studies (43–46%) (13, 14); (ii) we found a major difference in the shape of the curves across the different studies: in children from southwestern France the curve is linear with a small slope, as it is observed in adults without the presence of a plateau curve in older people; (iii) the difference in the kinetics could indicate differences in exposure to the virus, different transmission routes, and more marked environmental risk activities in the Corsican Island environment.

Our study has two limitations: (i) the samples corresponded to residual sera from patients having visited medical biology laboratories for blood sampling, which may have included a bias in the tested population. However, when we compared the results of the 18–19-yo patients (44.4%), the estimated seroprevalences were found to be very close to those determined in our previous study, 46.0 and 42.8% in different populations (university, blood donors, and patients from general practitioners); (ii) the lack of a questionnaire about the main risk factors.

## Conclusion

In conclusion, our study demonstrates that in the hyperendemic island of Corsica, (i) there is a homogeneous exposure of the population at the spatial level with no difference between genders; (ii) HEV exposure occurs from birth, resulting in 7.4% seropositivity at the age of 4 yo; (iii) super exposure is observed after the age of 9. Accordingly, specific studies should be conducted to determine the breadth of the situation identified in our study. The role of the environment and its contamination by domestic or wild swine excreta should be investigated using a One Health approach to reach conclusions that might be compared in different hot-spot regions. Indeed, it is questionable whether exposure to the swine reservoir alone can explain such high seroprevalences from an early age. Additional transmission routes beyond the zoonotic pathway are plausible. Thus, there could be various exposures, with known risk activities emerging at around 10 years of age, in addition to changes in diet and activities.

## Data availability statement

The raw data supporting the conclusions of this article will be made available by the authors, without undue reservation.

## Author contributions

LC: Conceptualization, Data curation, Formal analysis, Methodology, Writing – original draft. SM: Formal analysis, Writing – review & editing. NH: Formal analysis, Methodology, Writing – original draft. DD: Formal analysis, Methodology, Writing – review & editing. JC: Conceptualization, Investigation, Writing – review & editing. M-HS: Conceptualization, Investigation, Writing – review & editing. XL: Conceptualization, Methodology, Writing – review & editing. AF: Conceptualization, Methodology, Supervision, Writing – review & editing. RC: Conceptualization, Methodology, Supervision, Validation, Writing – review & editing.
